# Remodeling the Tumor Immune Environment in Breast Cancer via Bortezomib-based Combination Therapy

**DOI:** 10.7150/ijbs.122366

**Published:** 2025-09-06

**Authors:** Shaojuan Huang, Qiang Chen

**Affiliations:** 1 Department of Biomedical Sciences, Faculty of Health Sciences, University of Macau, Taipa, Macau SAR, China.; 2 Cancer Centre, Faculty of Health Sciences, University of Macau, Taipa, Macau SAR, China.; 3 MOE Frontier Science Centre for Precision Oncology, University of Macau, Taipa, Macau SAR, China.

**Keywords:** Bortezomib, TM, AMD3100, PSMB5, DNA damage, Tumor immune microenvironment

## Abstract

While proteasome inhibitors have revolutionized the treatment of hematologic malignancies and significantly improved patient survival, their efficacy in solid tumors remains limited. The recent work by Tang and colleagues demonstrates a novel combination strategy to overcome this limitation. Their study reveals that bortezomib, combined with either tetrathiomolybdate or AMD3100, synergistically kills breast cancer by downregulating expression of the proteasome subunit PSMB5. Crucially, the *in vivo* antitumor efficacy of these combinations is strictly dependent on an intact immune system, enabling cytotoxic CD8⁺ T cell responses. Although this study raises important mechanistic questions for future investigation, it significantly opens new avenues for expanding the therapeutic application of proteasome inhibitors in solid tumors.

Bortezomib, the first FDA-approved proteasome inhibitor, has long been utilized in hematologic malignancies such as multiple myeloma (MM).^1^ It exerts its anticancer effects through multiple mechanisms, including inhibition of the 26S proteasome, modulation of NF-κB activation, upregulation of pro-apoptotic factors, prevention of p53 degradation, and generation of reactive oxygen species. However, its application in solid tumors has been limited due to modest efficacy and significant side effects.^2^ Recent genome-wide screening revealed that enhanced proteasome activity contributes to broad drug resistance in breast cancer, and proteasome inhibition by bortezomib sensitizes resistant cells to chemotherapy.^3^ This suggests that combining proteasome inhibitors with other agents holds significant potential against solid tumors. Following this finding, Tang *et al.* identified two FDA-approved drugs, ammonium tetrathiomolybdate (TM, a copper chelator) and AMD3100 (CXCR4 inhibitor), which synergistically enhance bortezomib's killing effect on breast cancer *in vitro* and *in vivo* (Figure 1).^4^

In their study 4, *in vitro* drug screening of 115 known targeted drugs in *BRCA1*-mutant breast cancer cells revealed that TM and AMD3100 enhanced the cytotoxic effects of bortezomib. They further demonstrated that both compounds exacerbate proteasome dysfunction by suppressing the expression of PSMB5, a core β5 subunit of the 20S proteasome. Mechanistically, TM and AMD3100 disrupted mitochondrial function, reducing ATP production and subsequently activating AMP-activated protein kinase (AMPK). This activation blocked signal transducer and activator of transcription 3 (STAT3) phosphorylation, thereby downregulating PSMB5 expression. The combination of bortezomib with TM or AMD3100 induced synergistic effects via ATP/AMPK/STAT3/PSMB5 signaling axis, independent of *BRCA1* mutation status. While Tang *et al*. indicate potential efficacy against other solid tumors, the multifactorial mechanisms underlying bortezomib resistance and the heterogeneity of solid tumors require further investigation to define its precise therapeutic indications.^4^, ^5^ Notably, the study links AMPK activation to proteasome inhibition via STAT3 suppression, implying that strategies inducing AMPK activation may enhance bortezomib efficacy. However, previous study indicates that STAT3 activation induces expression not only of PSMB5 but also other β subunits of the 20S core complex, such as PSMB6 and PSMB7,^6^ which is not consistent with Tang *et al*.'s findings. Therefore, how AMPK-mediated STAT3 inhibition selectively regulates PSMB5 warrants further investigation to expand the clinical applicability.

Strikingly, the combination of bortezomib with TM or AMD3100 significantly inhibited breast cancer growth in immunocompetent mice but not in immunocompromised mice.^4^ This contrast highlights the important role of cytotoxic CD8⁺ T cell infiltration for therapeutic efficacy. Mechanistic studies revealed that the combinations induce DNA damage, activating the cyclic GMP-AMP synthase (cGAS)-stimulator of interferon genes (STING) pathway within cancer cells. This, in turn, triggers NF-κB signaling, leading to the upregulation of major histocompatibility complex class I (MHC I) molecules and C-C motif chemokine ligand 5 (CCL5). These upregulations promote CD8⁺ T cell recruitment and enhance their cytotoxic activity against cancer cells *in vivo*. While bortezomib alone exhibits limited efficacy against solid tumors *in vivo*, partly due to poor tumor penetration, its combination with TM or AMD3100 dramatically augments its antitumor effect - an enhancement strictly dependent on CD8⁺ T cells. Tang *et al*.'s work addresses a critical gap by elucidating how proteasome inhibition combined with TM/AMD3100 influences tumor growth *in vivo* through immune activation. It also highlights the importance of appropriate immunocompetent models for evaluating the therapeutic potential of antitumor agents effectively.

While Tang and his colleagues present a series of compelling findings, it also raises important questions for further investigation (Figure 1):

1. DNA damage-induced cGAS-STING activation *in vivo*: While the combination strategy activated the cGAS-STING pathway via DNA damage, leading to NF-κB activation, these findings were primarily obtained from *in vitro* models. Given the authors concluded that insufficient tumor drug accumulation limited efficacy in immunocompromised mice, it remains unclear whether the treatment induces adequate DNA damage to activate cGAS-STING *in vivo*. Although the authors plan to quantify intratumoral drug concentrations, two potential approaches could strengthen the *in vivo* validation: employing localized delivery (e.g., intratumoral injection) to enhance tumor-specific drug accumulation and assess potential efficacy improvements in immunocompromised mice; directly measuring DNA damage markers (e.g., γ-H2AX) and cGAS-STING activation, then evaluating whether cGAS-STING deficiency impedes the combination's efficacy *in vivo*.

2. BRCA1 status and synergistic effects: Proteasome inhibition has been reported to induce a “BRCAness” state in MM, depleting nuclear ubiquitin pools and impairing H2AX polyubiquitylation.^7^ Although the drug combinations showed synergistic effects regardless of BRCA1 status *in vitro*, the findings from *in vivo* models show the dependence on DNA damage-initiated cGAS-STING/NF-κB signaling. It suggests that *BRCA1*-mutant cancers might be more sensitive to this strategy. Investigating the involvement of BRCA1 and other DNA repair genes in this therapeutic strategy is warranted.

3. Impact on antigen presentation: CD8^+^ T cells play a critical role in anti-tumor immunity by recognizing antigens presented via MHC I molecules. As the proteasome is crucial for generating the antigenic peptides loaded onto MHC I, proteasome inhibitors can significantly impair antigenic peptide production.^8^ The findings from Tang *et al*. were primarily obtained using cancer cells overexpressing exogenous immunogenic proteins including green fluorescent protein, luciferase, ovalbumin (OVA). While OVA peptide presentation increased under drug combination treatment, the impact on endogenous tumor antigen presentation remains unclear. Investigating the immunopeptidome under combination treatment could help optimize dosing to minimize disruptions to antigen processing.

4. Comprehensive tumor microenvironment (TME) remodeling: Beyond directly targeting cancer cells, the drug combinations may also regulate other components of the TME. Tang *et al*. observed an increase in CD4^+^ memory T cells, which depend on MHC II molecules presented by antigen-presenting cells (APCs) such as macrophages, dendritic cells, and B cells. The CCL5/CCR5 axis has a dual function: it recruits CD8^+^ T cells to exert anti-tumor effects, but paradoxically, it facilitates tumor progression through multiple mechanisms.^9^ Additionally, AMD3100-mediated blockade of CXCR4, a receptor critical for fibroblasts-induced immunosuppression,^10^ could impact fibroblast infiltration and TME remodeling. Therefore, single-cell analysis of the TME could provide a comprehensive understanding of these complex effects.

In conclusion, Tang *et al.* employed a multifaceted strategy, integrating *in vitro* experiments, *in vivo* mouse models, and mechanistic studies to provide robust evidence for their conclusions. By demonstrating that combination of bortezomib with TM or AMD3100 enhances CD8^+^ T cell-mediated antitumor immunity, the study provides a strong rationale for repurposing these agents in solid cancer treatment. This work opens new avenues for enhancing the efficacy of immunotherapy in breast cancer and potentially other solid tumors using proteasome inhibitors. As research continues to unravel the complex interplay between cancer cells, the immune system, and the TME, such studies will be instrumental in advancing cancer immunotherapy.

## Figures and Tables

**Figure 1:**
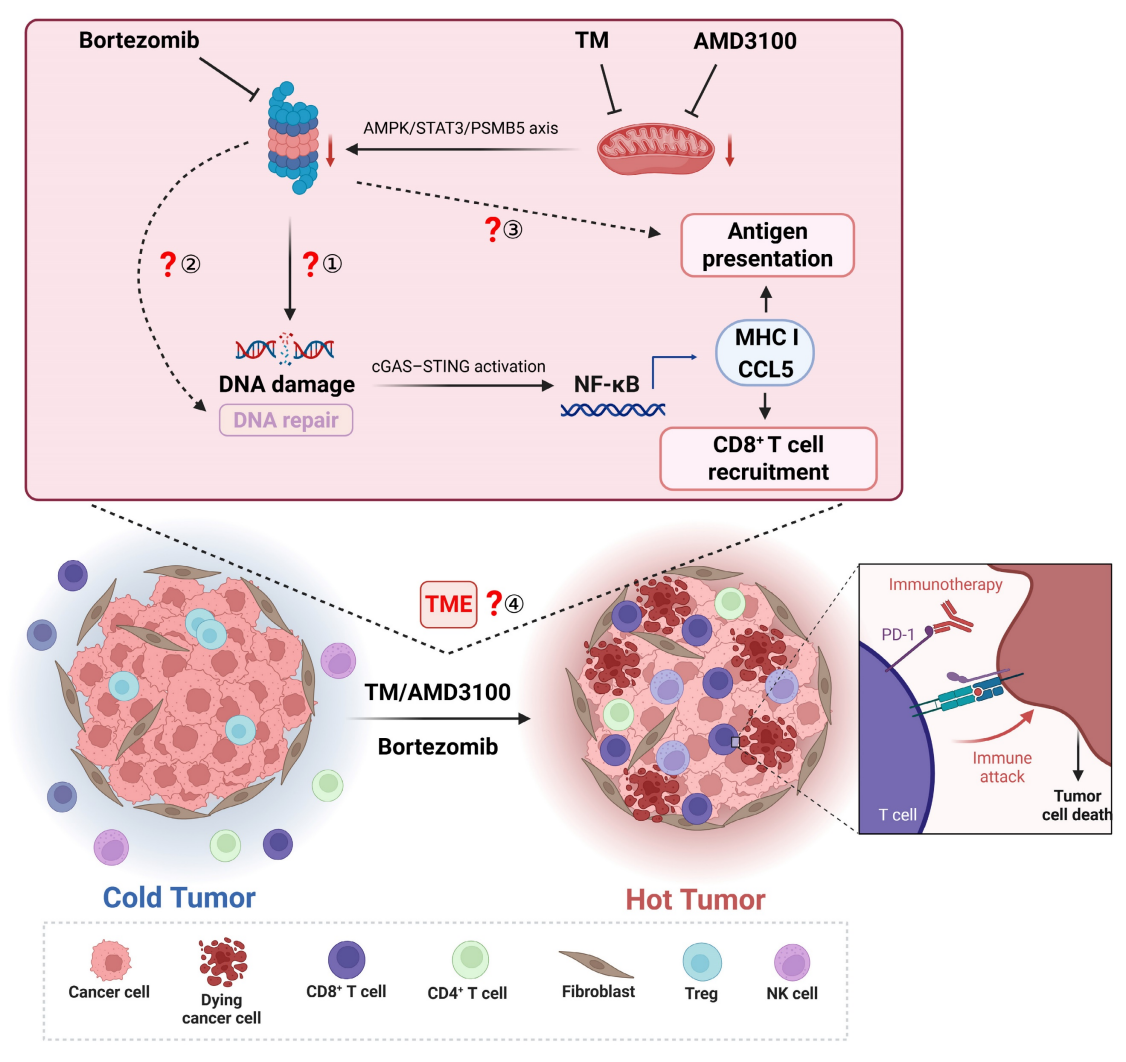
TM or AMD3100 synergizes with bortezomib to kill solid tumors via immune activation. TM or AMD3100 impairs mitochondrial function, resulting in proteasome dysfunction. Combination administration of TM or AMD3100 with bortezomib induces DNA damage, thereby activating the cGAS/STING pathway. This subsequently triggers NF-κB signaling, upregulating MHC class I-related genes and CCL5. Enhanced antigen presentation increases CD8+ T cell cytotoxicity, while CCL5 promotes their infiltration, collectively converting cold tumor into hot tumor. Created with BioRender.com.

## Abbreviations

FDA: Food and Drug Administration
PSMB5: Proteasome subunit beta type-5
MM: Multiple myeloma
NF-κB: Nuclear Factor Kappa-light-chain-enhancer of activated B cells
TM: Tetrathiomolybdate
CXCR4: C-X-C chemokine receptor type 4
BRCA1: Breast Cancer gene 1
AMPK: AMP-activated protein kinase
STAT3: Signal transducer and activator of transcription 3
PSMB6: Proteasome subunit beta type-6
PSMB7: Proteasome subunit beta type-7
cGAS: cyclic GMP-AMP synthase
STING: Stimulator of interferon genes
MHC I: Major histocompatibility complex class I
MHC II: Major histocompatibility complex class II
CCL5: C-C motif chemokine ligand 5
γ-H2AX: H2AX phosphorylated on serine 139
OVA: Ovalbumin
TME: Tumor microenvironment
APCs: Antigen-presenting cells
CCL5: Chemokine (C-C motif) ligand 5
CCR5: C-C chemokine receptor type 5
